# Enhanced spectral signatures with Ag nanoarrays in hyperspectral microscopy for CNN-based microplastics classfication

**DOI:** 10.3389/fchem.2025.1562743

**Published:** 2025-03-21

**Authors:** Xinwei Dong, Xu Zhao, Jianing Xu, Qianqian Chen, Hanwen Luo, Fuxin Zheng, Tao Zhang, Yansheng Liu

**Affiliations:** ^1^ Department of Joint Osteopathy, Liuzhou Worker’s Hospital, Liuzhou, China; ^2^ School of Electronic Engineering, Guangxi University of Science and Technology, Liuzhou, China; ^3^ School of Computer Science and Technology, Guangxi University of Science and Technology, Liuzhou, China

**Keywords:** microplastics, Ag nanoarrays, spectrum analysis, microscopic hyperspectral imaging, convolutional neural networks

## Abstract

Microplastics are a pervasive pollutant in aquatic ecosystems, raising critical environmental and public health concerns and driving the need for advanced detection technologies. Microscopic hyperspectral imaging (micro-HSI), known for its ability to simultaneously capture spatial and spectral information, has shown promise in microplastic analysis. However, its widespread application is hindered by limitations such as low signal-to-noise ratios (SNR) and reduced sensitivity to smaller microplastic particles. To address these challenges, this study investigates the use of Ag nanoarrays as reflective substrates for micro-HSI. The localized surface plasmon resonance (LSPR) effect of Ag nanoarrays enhances spectral resolution by suppressing background reflections and isolating microplastic reflection bands from interference. This improvement results in significantly increased SNR and more distinct spectral features. When analyzed using a 3D-2D convolutional neural network (3D-2D CNN), the integration of Ag nanoarrays improved classification accuracy from 90.17% to 98.98%. These enhancements were further validated through Support Vector Machine (SVM) analyses, demonstrating the robustness and reliability of the proposed approach. This study demonstrates the potential of combining Ag nanoarrays with 3D-2D CNN models to enhance micro-HSI performance, offering a novel and effective solution for precise microplastics detection and advancing chemical analysis, environmental monitoring, and related fields.

## 1 Introduction

One of the most crucial environmental issues currently is the ubiquitous presence of microplastics in aquatic ecosystems (Koelmans et al., 2022; Vethaak and Legler, 2021; Zhao et al., 2024; Li et al., 2023). Their persistence in the environment and the harmful impacts on organisms, including circulation throughout the body, penetration of vital barriers, and the potential to cause oxidative damage and immunological stress (Vethaak and Legler, 2021; Li et al., 2024; Kadac-Czapska et al., 2024). The World Wide Fund for Nature has reported that an average individual may ingest up to 5 g of microplastics each week (Nor et al., 2021). Despite the growing concern, accurately detecting and quantifying microplastics remains a formidable challenge (Yang et al., 2024; Rafiq and Xu, 2023). Traditional methods, such as Fourier-transform infrared spectroscopy (FTIR) (Campanale et al., 2023; Andoh et al., 2024), Raman spectroscopy (Fang et al., 2023; Sunil et al., 2024), and scanning electron microscopy (SEM) (Wang et al., 2017), rely on expensive equipment and time-consuming procedures that require skilled operators. Additionally, FTIR and Raman spectroscopy lack spatial resolution, while SEM lacks spectral information. These limitations hinder their scalability for high-throughput microplastic detection (Singh and Kumar, 2024). These limitations hinder the scalability of high-throughput microplastic detection, highlighting the urgent need for innovative and more efficient methodologies to address this critical issue.

Hyperspectral imaging (HSI), which captures both spatial and spectral information for each pixel (Bian et al., 2024), offers a promising solution for the rapid and accurate detection of microplastics in environmental samples (Ali et al., 2024; Serranti et al., 2024). Unlike traditional techniques, HSI can simultaneously deliver high-dimensional spatial and spectral data, making it highly advantageous for microplastic characterization and quantification (Yang et al., 2023a; Qu et al., 2022). This dual capability allows HSI to overcome the inherent limitations of conventional spectroscopic methods. However, the complexity and redundancy of HSI data have historically posed challenges for effective interpretation (Jaiswal et al., 2023). Fortunately, recent advancements in artificial neural network (ANN) algorithms have enabled the extraction of meaningful information from complex HSI datasets (Wambugu et al., 2021), making the combination of HSI and ANN highly effective for precise and accurate environmental monitoring (Jin et al., 2024; Hu et al., 2024).

Standard HSI techniques have a detection limit of around 200 μm, limiting their effectiveness for smaller microplastics (Peters et al., 2025; Sun et al., 2021; Padervand et al., 2020). Microscopic Hyperspectral Imaging (micro-HSI) offers the ability to detect microplastics smaller than 200 μm, providing high-resolution spatial and spectral information critical for environmental monitoring (Banu et al., 2023). However, under microscopic conditions, challenges such as strong background spectral reflections and localized optical effects, including enhanced scattering, absorption, and resonance phenomena, significantly hinder the quality of spectral signals (Rees et al., 2013; Bertani et al., 2013). These effects increase the complexity of light pathways, introduce additional noise, and obscure characteristic spectral features, thereby reducing the signal-to-noise ratio (SNR) (Paterova et al., 2020). Such challenges are especially pronounced in optically dense or heterogeneous samples, where scattering dominates the signal collection process. Addressing these limitations requires the development of advanced methodologies to improve spectral clarity and enhance detection accuracy, ensuring precise identification and characterization of microplastics at smaller scales (Yang et al., 2023b; Patil et al., 2022).

Silver (Ag) nanostructures exhibit an exceptional optical cross-section, resulting in a pronounced localized surface plasmon resonance (LSPR) effect in the visible light region (Mahmudin et al., 2024; Philip and Kumar, 2022; Liu and Huang, 2013). This unique property makes Ag nanoparticles widely utilized in surface-enhanced Raman spectroscopy (SERS), a technique renowned for its high sensitivity and selectivity in trace-level detection (Wu et al., 2021). The LSPR effect of Ag nanostructures is highly responsive to environmental changes, with shifts in resonance wavelength occurring due to variations in the refractive index of the surrounding medium (Montes-García et al., 2021). This sensitivity establishes Ag nanostructures as effective optical sensors capable of detecting subtle environmental changes (Kim et al., 2021; Xu and Geng, 2021). More importantly, the LSPR effect significantly enhances the scattering and absorption of visible light (Lv et al., 2022). In our experiments, When Ag nanostructures are used as substrates for reflection-mode micro-HSI, we observed a notable suppression of background reflection. This suppression appears to enhance spectral contrast, potentially aiding in the improved identification of chemical components. Such an enhancement is particularly valuable in micro-HSI applications, where precise spectral differentiation underpins effective material characterization.

Building on the unique optical properties of Ag nanostructures, this study explores their potential as substrate for micro-HSI to enhance spectral clarity and improve detection accuracy at smaller scales. To the best of our knowledge, the application of substrates with LSPR effects in reflection-mode micro-HSI for microplastic detection has not been systematically investigated. In this context, integrating Ag nanoarrays substrate with micro-HSI offers a promising strategy to address current detection limitations. Therefore, the objective of this study is to investigate the effectiveness of Ag nanoarrays substrates in enhancing the performance of reflection-mode micro-HSI for microplastic detection. Specifically, we seek to assess the influence of Ag nanoarrays on reducing background spectral reflections, enhancing the signal-to-noise ratio (SNR), and improving the precision of microplastic classification through the application of convolutional neural networks (CNN) and Support Vector Machines (SVM). This work aims to lay the foundation for a novel technological strategy that could advance microscopic hyperspectral detection applications.

## 2 Materials and methods

### 2.1 Chemicals and materials

Ultrapure silver (Ag, 99.999% purity) was kindly provided by Zhongnuo New Material (Beijing) Technology Co., Ltd. (Beijing, China). Microplastics with varying compositions, including polystyrene (PS), polyethylene terephthalate (PET), and polymethyl methacrylate (PMMA), were supplied by Polymer Plastic Co., Ltd., (Dongguan, China). The microplastic particles had an average diameter of approximately 1 μm, ensuring consistency in particle size for experimental analysis. High-quality silicon wafers (Si) were sourced from Jinan Huayao Optical Technology Co., Ltd., (Jinan, China). All chemical reagents used in the experiments were of analytical grade to ensure reliability and reproducibility of results. Solutions were prepared exclusively with deionized and decarbonated water to eliminate potential impurities that could interfere with the experiments.

### 2.2 Preparation of Ag nanoarrays

The preparation of silicon (Si) wafers began with a rigorous cleaning process to ensure a pristine surface. The wafers were immersed in a freshly prepared piranha solution, composed of concentrated sulfuric acid (98% H_2_SO_4_) and hydrogen peroxide (30% H_2_O_2_) in a 7:3 volume ratio. This highly oxidative solution was used to remove organic contaminants and increase the hydrophilicity of the wafer surface. The wafers were treated in the solution for 10 min, ensuring thorough cleaning. Following this step, the wafers underwent sequential rinsing with deionized water and ethanol, repeated three times to eliminate any residual chemicals.

Once the cleaning process was completed, the wafers were dried under a steady stream of nitrogen gas to prevent contamination from ambient particles. The cleaned and dried Si wafers were then carefully mounted in a thermal evaporation system for the deposition of Ag. Ultrapure silver target material (99.999%) was placed in an evaporation boat within the system. Using thermal evaporation, a uniform silver film with a thickness of approximately 15 nm was deposited onto the wafer surfaces. Subsequently, the deposited silver films underwent annealing in a muffle furnace at a temperature of 310°C for 47 min. Annealing significantly altered the surface energy of the Ag film, reorganizing atoms and transforming the uniform film into a well-ordered Ag nanoarrays.

### 2.3 Morphology characterization and Raman measurements

The surface morphology of the Ag nanoarrays was analyzed using a Hitachi S-4800 field emission (SEM), providing high-resolution imaging to assess the structural details of the nanostructures. For Raman spectroscopy analysis, a confocal micro-Raman system (Zolix, Beijing) was employed, utilizing a 532 nm excitation laser for spectral measurements. To ensure precise focus and high signal sensitivity, a 100× objective lens was used, paired with a diffraction grating of 1,800 lines/mm to achieve optimal spectral resolution. The laser power was carefully maintained at 0.5 mW to prevent any potential thermal damage to the sample, and the integration time for each Raman measurement was set to 1 s to balance signal clarity and efficiency.

### 2.4 Prepare datasets of microscopic hyperspectral imaging (micro-HSI)

Microplastic aqueous solutions were prepared at a concentration of 500 μg/mL using water as the solvent, reflecting the common practice of extracting microplastics from aquatic environments. To ensure uniform particle distribution, the solutions were subjected to ultrasonic dispersion using an ultrasonic bath operating at 40 kHz for 10 min. No additional dispersants were employed, as the study aimed to maintain consistency with the natural water-based conditions of microplastic samples. Following dispersion, 5 µL of the solution was carefully transferred onto the preprepared substrates using a micropipette for sample preparation. The samples were dried under an infrared lamp before undergoing microscopic hyperspectral imaging (micro-HSI) analysis.

To analyze the spectral and spatial properties of microplastic particles, hyperspectral imaging was employed using a benchtop hyperspectral imaging system (SM320, 3nh Inc., China) integrated with a CX40M microscope (Sunny Ltd., China). The microscope, equipped with a 500× magnification lens, enabled precise visualization of the microplastic samples. The HSI system operated across a spectral range of 400–1,000 nm with a high spectral resolution of 2 nm, ensuring the collection of detailed spectral profiles. Hyperspectral data were captured at a spatial resolution of 660 × 360 pixels, with spectral information recorded across 300 discrete wavelengths.

To evaluate the performance of the Ag nanoarrays substrates in hyperspectral analysis, micro-HSI data were collected for all microplastic samples on both Ag nanoarrays and Si substrates. The microplastic samples were classified into six distinct categories based on composition: pure PS, PET, PMMA, and three mixed groups (PS+PET, PS+PMMA, and PS+PET+PMMA). For each category, three hyperspectral images were acquired to ensure dataset consistency. Hyperspectral data from these images were segmented into 15 × 15 pixel regions, resulting in a total of 19,008 data samples for analysis, split equally between the two substrate types. For the river water samples, we replaced the deionized water with turbid river water as the solvent, while keeping all other procedures unchanged. This allowed us to obtain the microplastic hyperspectral dataset for the river water samples.

Data preprocessing involved two key steps: Savitzky-Golay (SG) smoothing to reduce noise and Z-score normalization to standardize the spectral data, ensuring consistency and comparability across all samples. The SG smoothing technique was applied to mitigate high-frequency noise inherent in hyperspectral measurements, which is particularly critical when characterizing subtle optical enhancements induced by Ag nanoarrays. The processed datasets were evenly split into two subsets, with 50% allocated for training the model and the remaining 50% reserved for testing. These preprocessed datasets were then used to train and validate classification models, including a convolutional neural network (CNN) and a support vector machine (SVM).

### 2.5 Introduction to the hybrid 3D-2D CNN model architecture

To address the spectral-spatial complexity of hyperspectral imaging (HSI) data in microplastic characterization, a hybrid 3D-2D convolutional neural network (CNN) was developed. This architecture synergizes the strengths of 3D convolutions for joint spectral-spatial feature extraction and 2D convolutions for efficient spatial representation learning, making it particularly suitable for analyzing microplastic HSI datasets acquired from Ag nanoarrays substrates.

**FIGURE 1:**
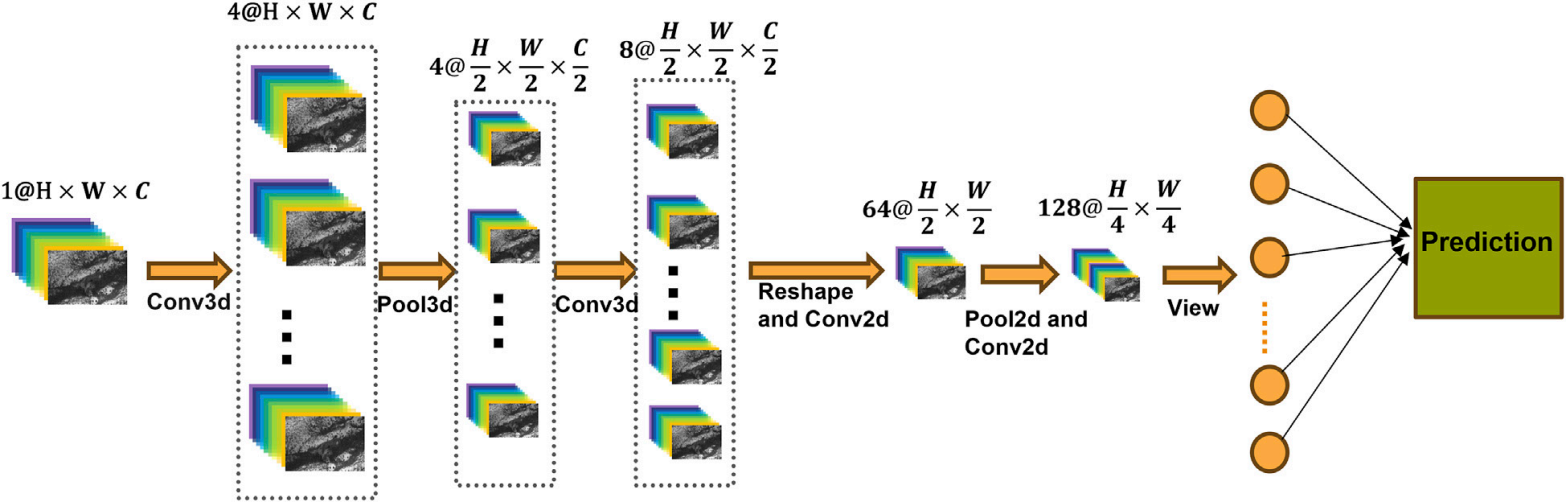
Overall architecture of the hybrid 3D-2D CNN model.

The proposed model architecture, illustrated in Figure 1, begins with 3D convolutional layers that process the input hyperspectral data, characterized by dimensions 1@H × W × C. Here, 1 denotes the number of input channels, H and W represent the spatial dimensions (height and width), and C signifies the spectral dimension. This initial stage facilitates the concurrent extraction of spatial-spectral features (Li et al., 2017). Following the 3D convolutional layers, 3D pooling is applied, reducing the dimensions to H/2 × W/2 × C/2 while retaining essential spatial-spectral information. After an additional 3D convolutional layer, the 3D feature tensor is reshaped into a 2D representation (K@H′ × W′) through depth-to-space transformation, where K denotes the synthesized feature channels. Then the 2D convolutions are applied to enhance the extraction of deeper spatial features while reducing computational complexity (Fırat et al., 2022). Progressive pooling layers continue to downsample the data, culminating in fully connected layers that perform the final classification. The network utilizes cross-entropy loss as the objective function to accomplish a six-class classification task. The 3D-2D CNN model was implemented using the PyTorch framework and simulated on a high-performance computer equipped with a GTX 4090 graphics card.

## 3 Results and discussion

### 3.1 Materials characterization and spectral comparative analysis

**FIGURE 2:**
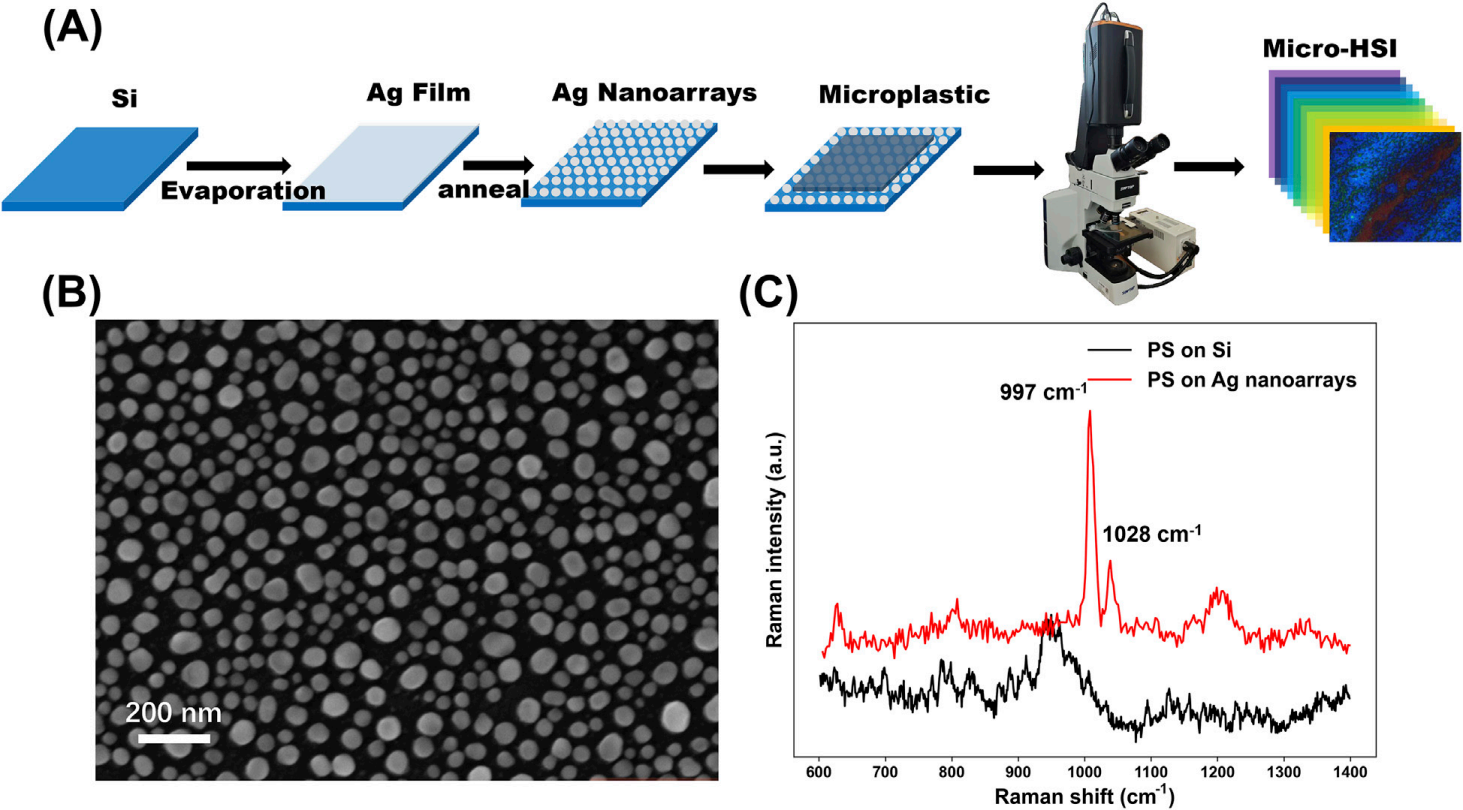
**(A)** Schematically illustrates the complete experimental workflow for acquiring micro-HSI data of microplastics; **(B)** High-magnification SEM image of the Ag nanoarray substrates; **(C)** Raman spectra of PS microplastic samples on Ag nanoarray substrates were compared with those on Si substrates.

Figure 2A illustrates the experimental workflow for acquiring micro-HSI data. The process begins with the deposition of Ag film onto Si substrates via thermal evaporation. The deposited film is subsequently subjected to thermal annealing, resulting in the formation of well-ordered Ag nanoarrays. A microplastic solution is then applied to the nanoarrays substrates and allowed to dry. Micro-HSI data are collected using a hyperspectral imaging system integrated with an optical microscope, enabling high-resolution spatial and spectral analysis of the samples. Figure 2B shows a high-magnification scanning electron microscopy (SEM) image of the fabricated Ag nanoarrays, revealing that the nanoarrays is composed of nanocolumns with diameters ranging from 30 to 100 nm. A low-magnification SEM image (Supplementary Figure S1) further confirms that the Ag nanoarrays substrate features a relatively smooth and uniform surface. To further illustrate the formation of the Ag nanoarrays, we have included two additional SEM images (Supplementary Figure S2) comparing the morphology before and after annealing at the same magnification in the revised Supplementary Material. After annealing, the Ag nanostructure clearly transitions from a film with numerous channels to a well-ordered nanoarray morphology. This controlled morphology and consistent size distribution are critical for generating LSPR effect, which plays a key role in enhancing optical signals for micro-HSI.

Figure 2C presents the Raman spectra of polystyrene (PS) microplastic samples deposited on Ag nanoarrays substrates compared to those on Si substrates. The PS samples on Ag nanoarrays exhibit sharp and well-defined characteristic peaks, such as the symmetric ring breathing mode at 997 cm^−1^ and the CH in-plane deformation mode at 1,028 cm^−1^ (Zhou et al., 2021; Mikac et al., 2023). In contrast, PS samples on Si substrates display negligible Raman signals, highlighting the superior signal enhancement provided by the Ag nanoarrays. This remarkable enhancement in Raman intensity is attributed to the LSPR effect induced by the Ag nanoarrays (Li et al., 2020). The LSPR amplifies the local electromagnetic field at the substrate surface, significantly boosting the Raman scattering efficiency of the PS molecules (Li et al., 2020). These findings provide strong preliminary evidence of the robust LSPR effect generated by the fabricated Ag nanoarrays, underscoring its potential to enhance spectroscopic techniques for micro-HSI.

**FIGURE 3:**
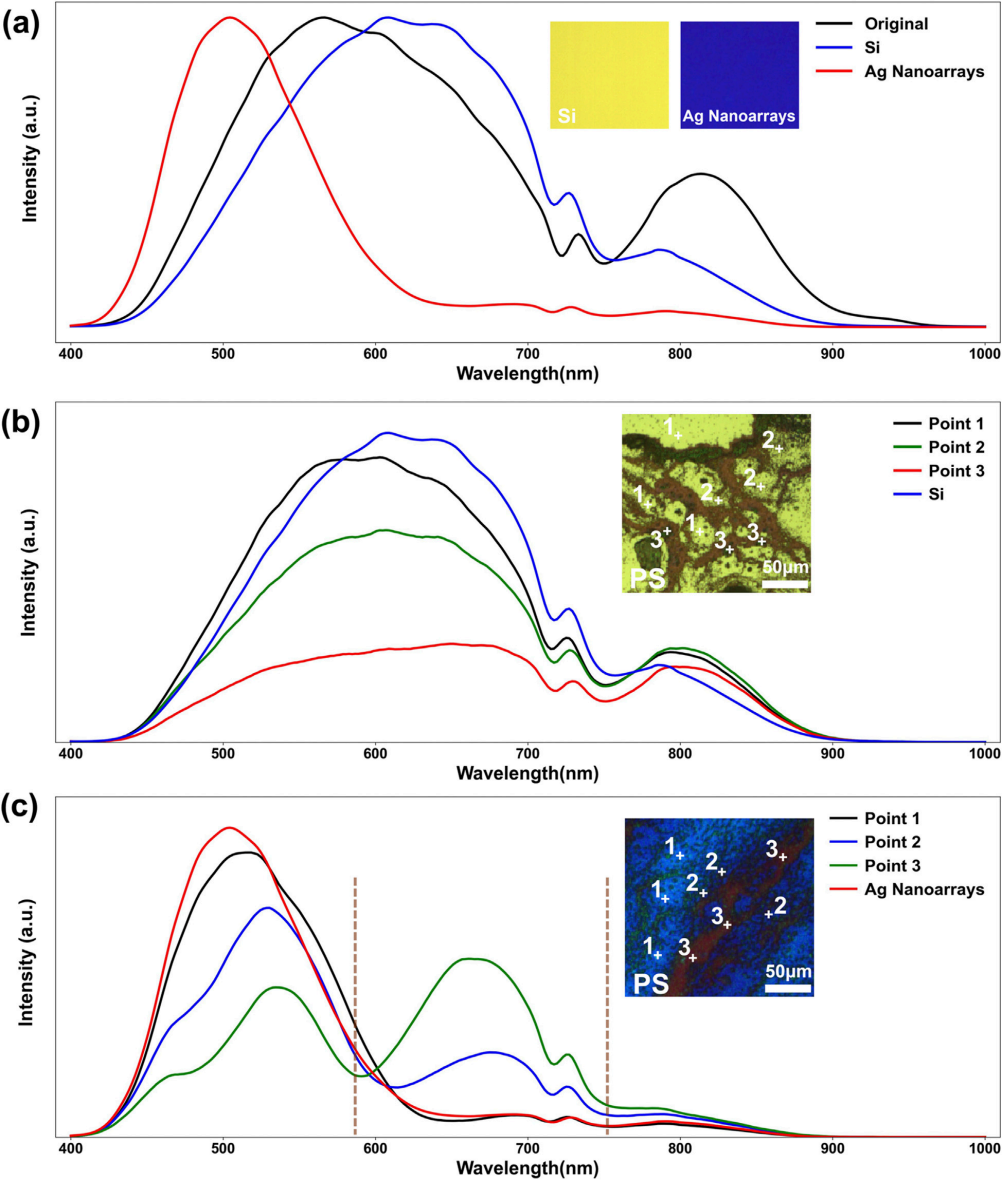
**(a)** A comparative analysis of the spectrum of the Micro-HSI system’s original light source, the average reflection spectra of Ag nanoarray substrate and Si substrate; The average reflectance spectra obtained from micro-HSI at three representative locations on dried PS microplastic particles, along with the reflectance spectra of their respective substrates: **(b)** Si substrate and **(c)** Ag nanoarray substrate. The insets in **(b, c)** provide 500× morphological images of the dried PS particles on Si and Ag nanoarray substrates. Points 1, 2, and 3 correspond to regions with low, moderate, and high particle densities, respectively.

Reflection-mode micro-HSI spectral data often suffer from strong background reflection (Durkan and Shvets, 1998), prompting this study to investigate the influence of Ag nanoarrays as micro-HSI substrates on reflection spectra. Figure 3a compares the raw spectrum of the hyperspectral light source with the reflection spectra of Si and Ag nanoarrays substrates. The reflection spectrum of the Si substrate closely resembles the light source spectrum, with a broad reflection band spanning 400–900 nm and a peak around 620 nm. In contrast, the Ag nanoarrays reflection spectrum exhibits a much narrower reflection band, primarily concentrated in the 400–580 nm range, with a peak at approximately 490 nm. The inset in Figure 3a visually highlights the differences between the two substrates under micro-HSI, displaying pseudo-three-channel color images (at wavelengths of 490 nm for blue, 590 nm for green, and 660 nm for red). The Si substrate appears warm yellow, resembling the halogen light source, while the Ag nanoarrays substrate shows a distinct deep blue color. The Ag nanoarrays reflection spectrum demonstrates a significant reduction in reflected light intensity for wavelengths above 580 nm compared to the light source spectrum. This reduction is likely attributed to the LSPR effect of the Ag nanoarrays, which enhances scattering and absorption in the 500–900 nm range, thereby greatly diminishing the reflection intensity. Compared to the Si substrate, the weak background reflection above 580 nm provided by the Ag nanoarrays substantially reduces the overall background noise, leading to a significant improvement in the SNR of the sample’s reflection spectra. To evaluate the long-term stability of the Ag nanoarrays, we examined their micromorphology and optical properties after a 4-month interval. The results (Supplementary Figure S3A) reveal no significant changes in the morphology of the Ag nanoarrays. Furthermore, their performance (Supplementary Figure S3B) as substrates for micro-HSI remains stable, consistently reducing reflection intensity in the 500–900 nm range.

To further investigate the impact of Ag nanoarrays substrate on microplastic sample imaging using micro-HSI, six distinct categories of microplastics based on their composition were experimentally analyzed: pure polystyrene (PS), polyethylene terephthalate (PET), polymethyl methacrylate (PMMA), and three mixed groups (PS and PET, PS and PMMA, and PS, PET, and PMMA). Supplementary Figure S4 shows high-magnification SEM images of all the microplastics, with the majority of their morphologies being spherical or ellipsoidal, and sizes ranging from 0.5 to 1 μm. Figures 3b, c present high-magnification micro-HSI reflection spectra of dried PS microplastic samples on Si substrates and Ag nanoarrays substrates, respectively. The insets in Figures 3b, c display morphological photographs (500×) of the dried PS particles on Si and Ag nanoarrays substrates. The approximately 1 μm-sized particles are relatively dispersed, forming regions with varying densities. The spectral curves of Points 1, 2, and 3 in the figure represent the averaged curves taken from three different points, corresponding to areas with low, moderate, and high particle density of the sample, respectively.

On the Si substrate (inset in Figure 3b), the colors of these three points transition from yellow-green to orange as the particle density increases. In contrast, on the Ag nanoarrays substrate (inset in Figure 3c), the colors shift from deep blue to green and then to deep red with increasing particle density, demonstrating a significantly stronger color contrast. Comparative analysis of the reflection spectra for these three points reveals that on the Si substrate (Figure 3b), the reflection bands across all points closely resemble the Si substrate’s reflection spectra, spanning the entire 400–900 nm range. Consequently, the microplastic reflection bands overlap with the background, making the spectral information of the microplastics difficult to discern. Conversely, on the Ag nanoarrays substrate (Figure 3c), the reflection spectra of the PS microplastic samples exhibit a distinct separation, with reflection band primarily concentrated between 580 and 750 nm. The peak of the reflection band occurs around 670 nm, clearly distinct from the background reflection band of the Ag nanoarrays substrate. For the highest particle density point (Point 3), the reflection spectrum distinctly shows the substrate’s background reflection band in the 400–580 nm range and the PS sample’s reflection band in the 580–750 nm range. This separation significantly reduces the interference from background reflections, thereby enhancing the SNR of the microplastic spectral information. As a result, the imaging effect achieved with Ag nanoarrays substrate resembles dark-field imaging but offers greater signal clarity (Zheng et al., 2022; Liu et al., 2021). Supplementary Figures S5–S9 demonstrate similar spectral enhancements for the other five microplastic categories, further validating the superiority of Ag nanoarrays substrates. These findings highlight the effectiveness of Ag nanoarrays in enhancing spectral clarity and detection accuracy in micro-HSI, providing a promising approach for precise microplastic identification and characterization.

**FIGURE 4:**
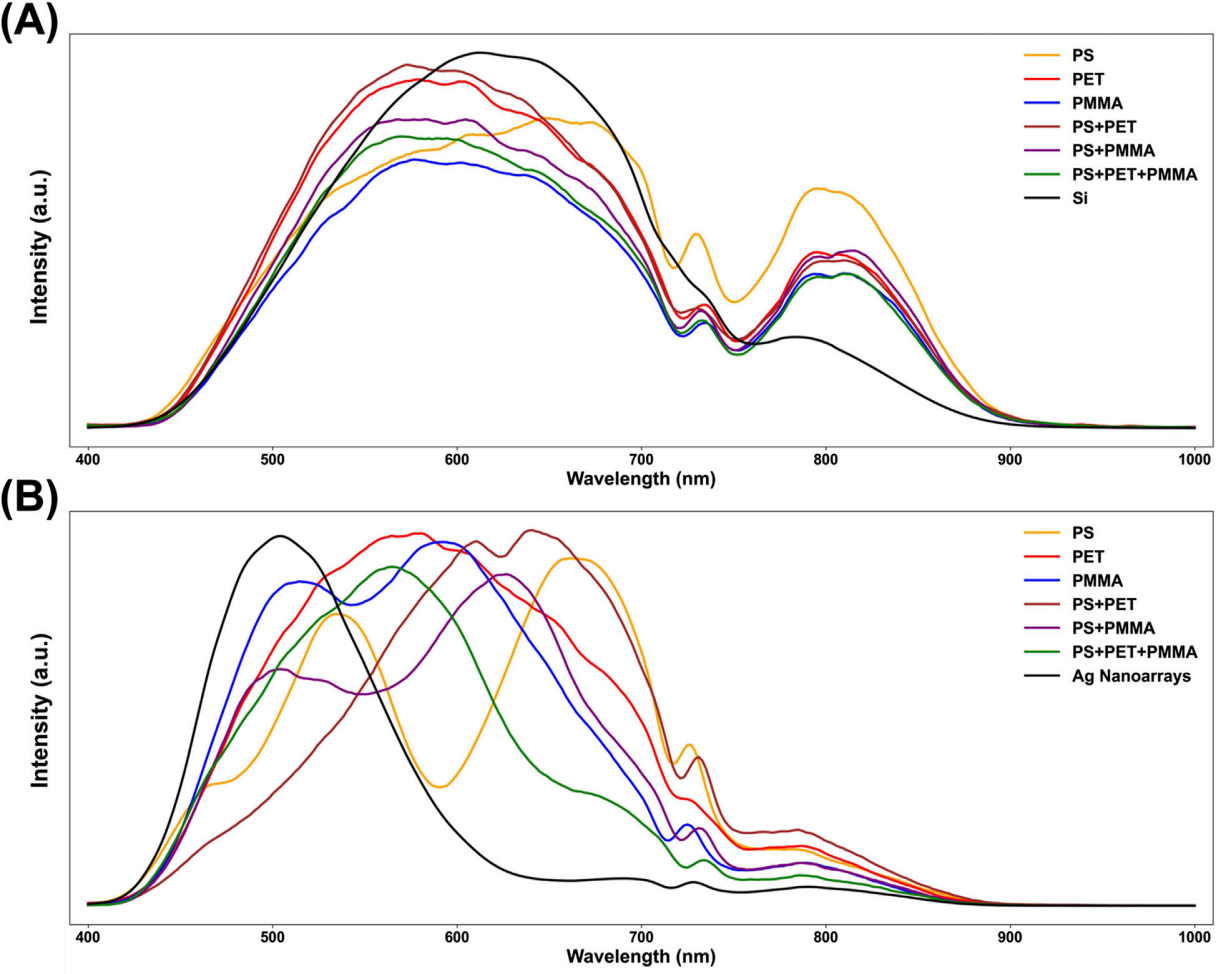
The averaged reflection spectra of three distinct points for six different microplastic categories and their corresponding substrates on **(A)** Si substrates and **(B)** Ag nanoarray substrates.

To compare the reflection spectra of six distinct microplastic categories on Si substrate and Ag nanoarrays substrate, Figures 4A, B present the respective averaged spectral curves. On the Si substrate (Figure 4A), the reflection bands of all six microplastic categories span the entire 400–900 nm range, closely overlapping with the background reflection bands of the pure Si substrate. This overlap results in similar spectral features across the six samples, making differentiation challenging. In contrast, for microplastics deposited on Ag nanoarrays, the influence of the substrate’s background reflection is significantly diminished. Consequently, the reflection spectra (Figure 4B) of the six microplastic categories exhibit distinct spectral features. Each sample displays unique reflection band distributions, with peak wavelengths ranging from 560 nm to 670 nm. This differentiation is advantageous for subsequent neural network-based feature extraction and classification. Furthermore, the LSPR effect of the Ag nanoarrays is highly sensitive to the refractive index of the surface-adsorbed samples (Montes-García et al., 2021). Variations in the refractive index among different microplastic samples result in distinct shifts in the resonance wavelength, thereby producing unique reflection spectra for each sample type. Overall, the comprehensive spectral comparison analysis clearly demonstrates that Ag nanoarrays amplify spectral features and enhance the overall SNR. This highlights the potential of Ag nanoarrays substrate for micro-HSI applications, underscoring their effectiveness in improving detection accuracy and spectral clarity for microplastic identification.

### 3.2 Ablation study of the hybrid 3D-2D CNN model

The architecture of the proposed hybrid 3D-2D CNN model seamlessly integrates 3D and 2D convolutional layers to effectively harness both spatial and spectral features from hyperspectral imaging data. Hyperspectral imaging inherently encapsulates a wealth of spectral and spatial information, demanding a model adept at capturing these dual dimensions. An ablation study, presented in the Supplementary Material (Supplementary Figure S10), was conducted to evaluate the contributions of individual network components. Models utilizing only 3D CNNs were capable of capturing joint spectral-spatial features but lacked the refined spatial processing achievable with 2D CNNs. Conversely, models employing solely 2D CNNs failed to fully leverage the rich spectral information inherent in hyperspectral data, resulting in reduced classification accuracy. The hybrid 3D-2D CNN architecture demonstrated superior performance compared to both single-dimension models, highlighting the critical role of integrating both convolutional dimensions for optimal feature representation.

This hybrid 3D-2D CNN architecture leverages the strengths of both convolutional approaches (Feng et al., 2019). While traditional 2D CNNs excel at extracting spatial features from RGB images with limited channels, hyperspectral images contain hundreds of spectral bands, each providing unique spectral information. Standard 2D CNNs are insufficient for fully integrating the rich spatial-spectral data inherent in HSI. By incorporating 3D convolutional layers at the initial stages, the model effectively captures the intricate relationships between different spectral bands and spatial locations, facilitating more comprehensive feature learning. This integration of advanced substrate technology with a tailored CNN architecture underscores the potential of Ag nanoarrays in enhancing micro-HSI for precise microplastic detection and characterization.

### 3.3 Classification performance analysis

**FIGURE 5:**
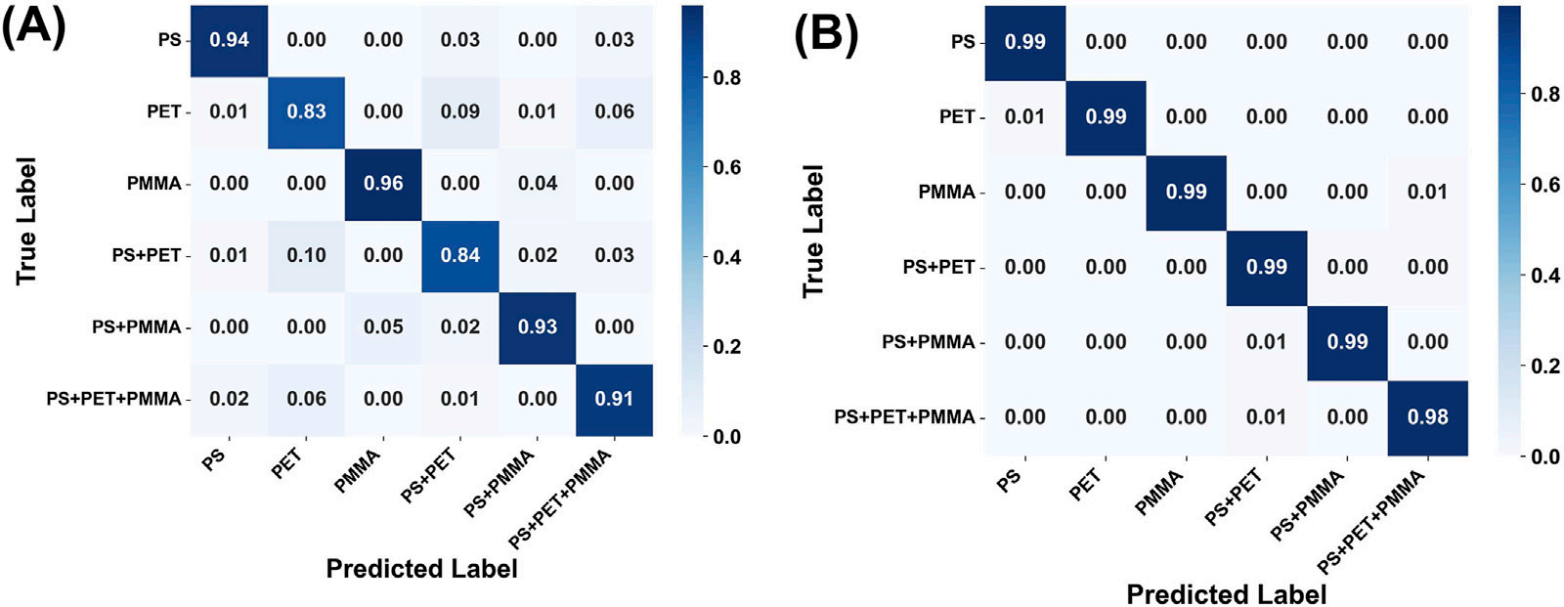
Confusion matrices of the hybrid 3D-2D CNN model applied to Micro-HSI datasets obtained from **(A)** Si substrates and **(B)** Ag nanoarray substrates.

To further evaluate the advantages of Ag nanoarray substrates, micro-HSI datasets obtained from both Si and Ag nanoarray substrates were analyzed using the proposed hybrid 3D-2D CNN model for feature learning and six-class classification. Supplementary Figures S11, S12 present the training loss curves and test accuracy curves for the training process of datasets based on the Si and Ag nanoarrays substrates, respectively. Figure 5 shows the confusion matrices for the classification performance of the hybrid 3D-2D CNN model on micro-HSI data from Si substrates (Figure 5A) and Ag nanoarrays substrates (Figure 5B). The confusion matrices compare the true labels of the six microplastic types, against the predicted labels by the model (Theissler et al., 2022). Each matrix cell represents the proportion of samples correctly or incorrectly classified, with values closer to 1.0 indicating higher classification accuracy.

In Figure 5A, the classification performance of the micro-HSI dataset on Si substrates shows relatively high accuracy (91%–96%) for most microplastic types, except for PET and PS+PET, which achieved lower accuracies of 83% and 84%, respectively. The overall six-class average accuracy for Si substrates is 90.17% (Table 1). These results suggest that while the hybrid 3D-2D CNN model can extract spatial and spectral features from the Si-based micro-HSI dataset, its classification performance is limited by the relatively low SNR and overlapping spectral features of the microplastic samples on Si substrates. As discussed earlier, the micro-HSI dataset on Ag nanoarrays substrates demonstrates significant advantages in terms of the SNR of the microplastic reflection spectra. This enhancement greatly facilitates the feature extraction and analysis capabilities of the 3D-2D CNN model, ultimately leading to superior classification performance. Indeed, when the micro-HSI dataset based on Ag nanoarrays substrates is used, the classification performance of the 3D-2D CNN model improves markedly, as shown in Figure 5B. The classification accuracy increases significantly across all categories, particularly for mixed microplastic samples. Individual microplastic types, including PS, PET, and PMMA, achieve near-perfect accuracy values of 0.99 or higher. Similarly, mixed classes such as PS+PET and PS+PMMA attain an accuracy of 0.99, while the more complex PS+PET+PMMA class achieves an accuracy of 0.98. Table 1 provides a detailed comparison of performance metrics, highlighting the overall superiority of Ag nanoarrays substrates. The dataset based on Ag nanoarrays achieves an overall classification accuracy of 98.98%, with precision, recall, and F1-scores all approximately 99%. In contrast, the dataset based on Si substrates achieves an accuracy of 90.17%, with precision, recall, and F1-scores around 90%. Notably, the error rate of the Si substrate-based dataset is nearly 10 times higher than that of the Ag nanoarrays substrate-based dataset. This comparison underscores the critical role of Ag nanoarrays substrates in enhancing hyperspectral data quality and classification performance.

**TABLE 1:** Comparison of the classification performance metrics for 3D-2D CNN and SVM models applied to micro-HSI datasets from samples on Ag nanoarrays and Si Substrates.

Model	Substrate	Accuracy	Recall	F1 score	Precision
3D-2D-CNN	Ag nanoarrays	98.98%	98.98%	98.98%	98.99%
	Si	90.17%	90.17%	90.16%	90.17%
SVM	Ag nanoarrays	94.95%	94.95%	94.96%	95.01%
	Si	72.58%	72.58%	72.34%	72.32%

The improved performance is attributed to the LSPR effects of the Ag nanoarrays. The LSPR effect enhances the scattering and absorption of light in the 550–900 nm range, significantly reducing the substrate’s background reflection intensity (Lv et al., 2022). Without the interference of background reflection spectra, the reflection spectral information beyond 580 nm is primarily attributed to the microplastic samples themselves (as shown in Figure 3c). This greatly increases the SNR and provides richer spectral features. Additionally, the LSPR effect exhibits high sensitivity to variations in the refractive index of materials in surface. This sensitivity can further contribute to distinct spectral features between different microplastic types, enhancing the model’s ability to classify samples accurately. In summary, the Ag nanoarrays substrate not only suppresses background reflection but also amplifies microplastic spectral features, providing a robust platform for micro-HSI data acquisition. Combined with the hybrid 3D-2D CNN model, this approach offers a powerful method for precise and reliable classification of microplastic samples, demonstrating its potential for advanced environmental monitoring and detection applications.

**FIGURE 6:**
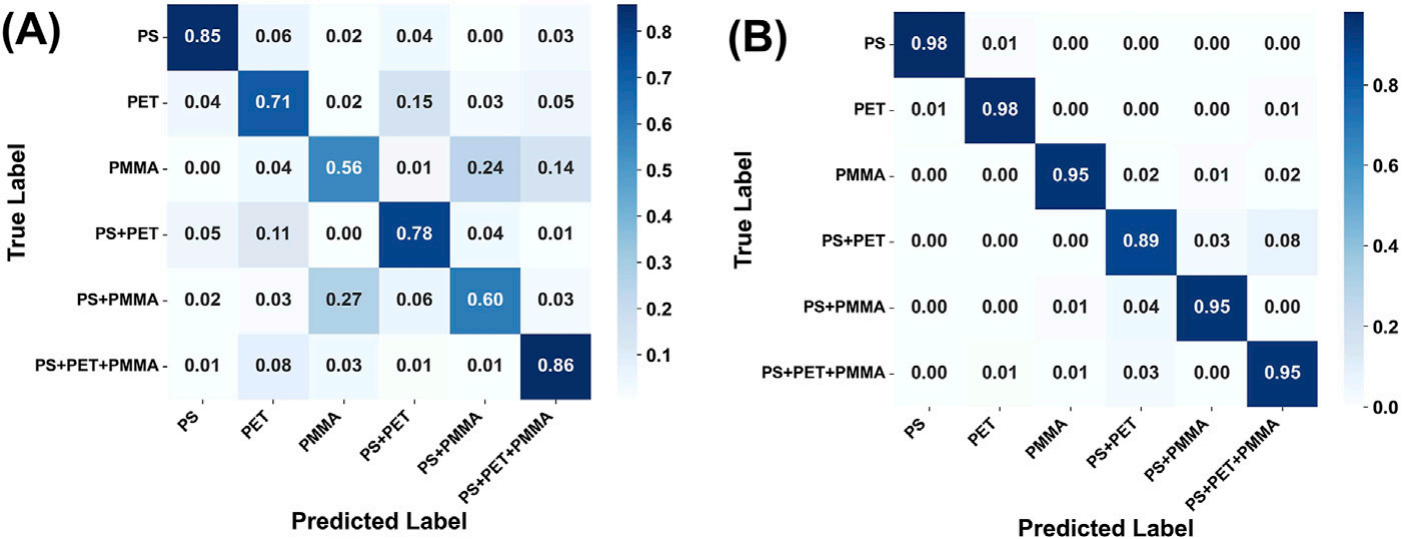
Confusion matrices of the SVM applied to micro-HSI datasets obtained from **(A)** Si substrates and **(B)** Ag nanoarray substrates.

Support Vector Machine (SVM) is a classical machine learning algorithm widely used for classification tasks. It is known for its ability to find an optimal hyperplane in high-dimensional space, maximizing the separation between classes (Quadir and Tanveer, 2024). In this study, SVM was employed as a complementary baseline model alongside the 3D-2D CNN to further validate the advantages of Ag nanoarrays substrates in enhancing micro-HSI spectral features. The SVM classification results (Figure 6; Table 1) show a significant improvement in performance when using Ag nanoarrays-enhanced datasets compared to those based on pure Si substrates. The Ag nanoarrays-enhanced datasets (Table 1) achieved an overall accuracy of 94.95%, substantially higher than the 72.58% accuracy observed with Si substrates. Moreover, the precision, recall, and F1-scores for Ag nanoarrays-based samples consistently approach 0.95, whereas the corresponding metrics for Si substrate-based samples were markedly lower. These results highlight that the improved spectral features facilitated by the Ag nanoarrays not only benefit advanced deep learning models such as the CNN but also enhance the performance of traditional classifiers like SVM. This further validates the effectiveness of the LSPR effect in improving the discriminative power of micro-HSI data. However, the SVM classification performance on both substrates was inferior to that of the 3D-2D CNN model, underscoring the superior capability of CNNs in analyzing the complex spatial-spectral features of micro-HSI data. The CNN model’s ability to jointly extract and learn intricate spatial and spectral relationships provides a distinct advantage over traditional classifiers, particularly for high-dimensional hyperspectral data.

Both the SVM and 3D-2D CNN models demonstrated a consistent trend: the Ag nanoarrays substrate significantly enhanced classification performance across all metrics. This underscores the efficacy of Ag nanoarrays in overcoming the limitations of traditional micro-HSI methods, advancing the precision and reliability of environmental monitoring techniques. By combining Ag nanoarrays-enhanced spectral data with advanced machine learning models, this approach offers a robust and promising framework for the detection and classification of microplastics. We also compared silver nanoarray-enhanced micro-HIS with Raman spectroscopy for microplastic detection. While Raman spectroscopy (Supplementary Figure S13) showed limited signal detection for microplastics such as PS and PET and encountered challenges with other samples, this highlights the advantages of the approach proposed in this study. To further evaluate the practical applicability of the proposed strategy in real-world aquatic environments, we conducted additional experiments using river water as the solvent for microplastic samples. As illustrated in Supplementary Figure S14, the classification performance significantly declined due to interference from a mix of environmental impurities of various sizes. The overall accuracy of the 3D-2D CNN model dropped to 80.64%, down from 98.98% in purified environments. These results highlight the limitations of the current approach in complex water conditions, where environmental noise impacted detection performance. Moving forward, optimizing the Ag nanoarrays and enhancing the AI model for improved computational efficiency will be critical for real-time monitoring applications.

## 4 Conclusion

In this study, we demonstrated the significant advantages of Ag nanoarrays substrates in enhancing the detection and classification of microplastic samples using reflection-mode micro-hyperspectral imaging (micro-HSI). Compared to conventional Si substrates, Ag nanoarrays provided improved signal-to-noise ratios (SNR) and richer spectral features, attributable to the localized surface plasmon resonance (LSPR) effect. The LSPR effect enhanced scattering and absorption, effectively suppressing background reflection in the 550–900 nm wavelength range and separating the microplastic reflection bands from the background. These enhanced spectral features were utilized in six-class classification tasks employing a hybrid 3D-2D CNN model. The micro-HSI dataset based on Ag nanoarrays consistently outperformed its Si substrate counterpart across all evaluation metrics, achieving an accuracy of 98.98% compared to 90.17% for the Si substrate, with error rates decreasing by nearly 90%. Additionally, traditional machine learning models, such as support vector machines (SVM), exhibited significant improvements in classification accuracy when utilizing Ag nanoarrays-enhanced data.

This study is the first to employ a plasmonic substrate in reflection-mode micro-HSI to separate microplastic reflection bands from background spectra by effectively suppressing background reflection. Furthermore, our findings highlight the synergistic effect between Ag nanoarrays and CNN models, demonstrating the ability of Ag nanoarrays to enhance micro-HSI data quality for precise feature extraction and classification. These advancements pave the way for broader applications of plasmonic-based micro-HSI in microscopic detection and analysis across chemistry, biomedicine, and materials science.

## Data Availability

The raw data supporting the conclusions of this article will be made available by the authors, without undue reservation.
