# Supplementary material for: Enhanced spectral signatures with Ag nanoarrays in hyperspectral microscopy for CNN-based microplastics classfication
Source: Front Chem. 2025 Mar 21;13:1562743. doi: 10.3389/fchem.2025.1562743 (PMC11968667; doi:10.3389/fchem.2025.1562743)
Supplement: Supplementary file 1 [file DataSheet1.pdf]

Supplementary Material

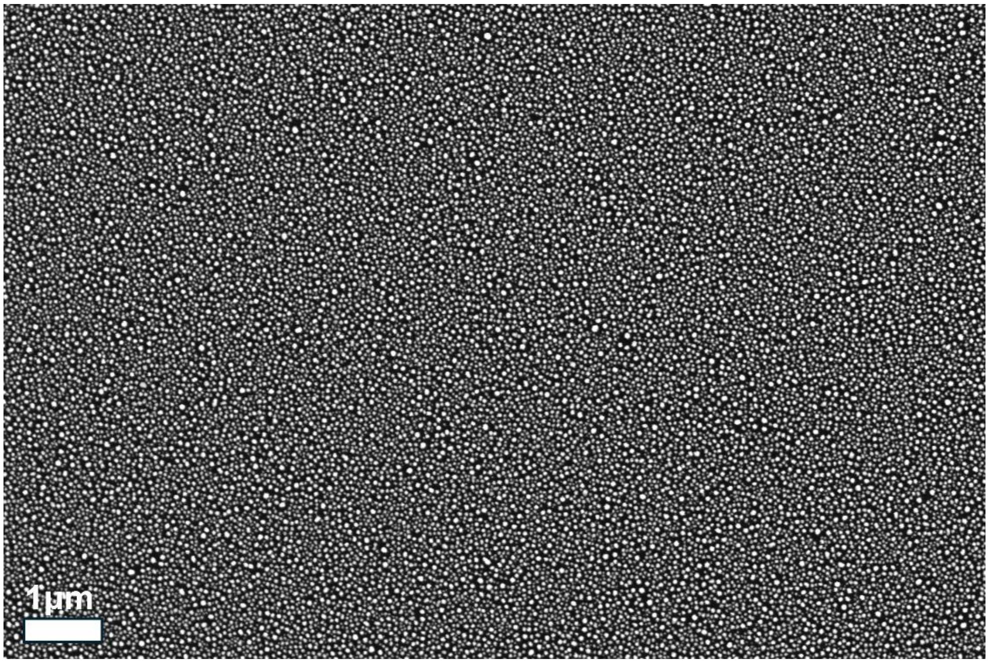

Figure S1. Low-magnification SEM image of Ag nanoarrays substrates

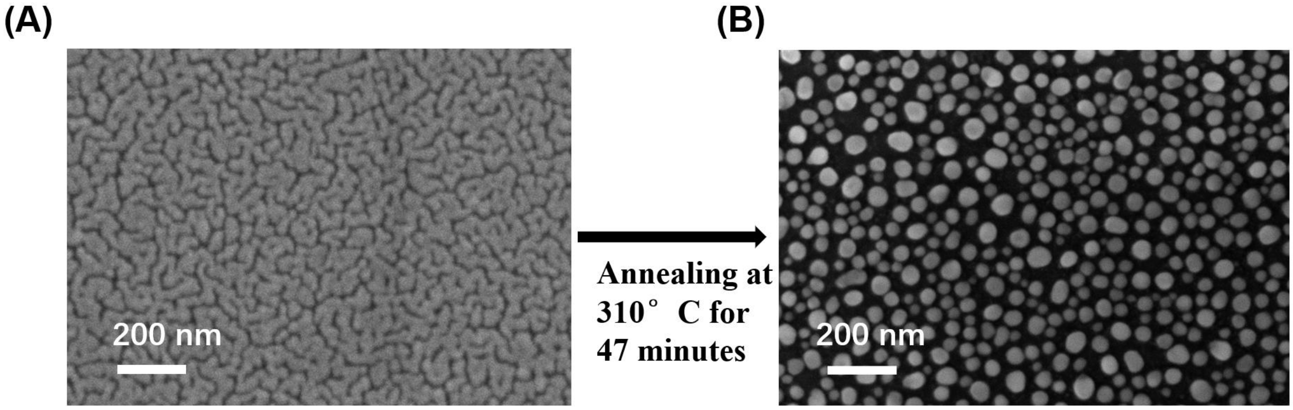

Figure S2. High-magnification SEM images of Ag nanostructures (A) before and (B) after annealing at 310°C for 47 minutes

To further illustrate the formation of the Ag nanoarrays, we have included two additional SEM images (Figure S2) comparing the morphology before and after annealing at the same magnification in the revised supplementary materials. After annealing, the Ag nanostructures clearly transition from a film with numerous channels to a well-ordered nanoarray morphology.

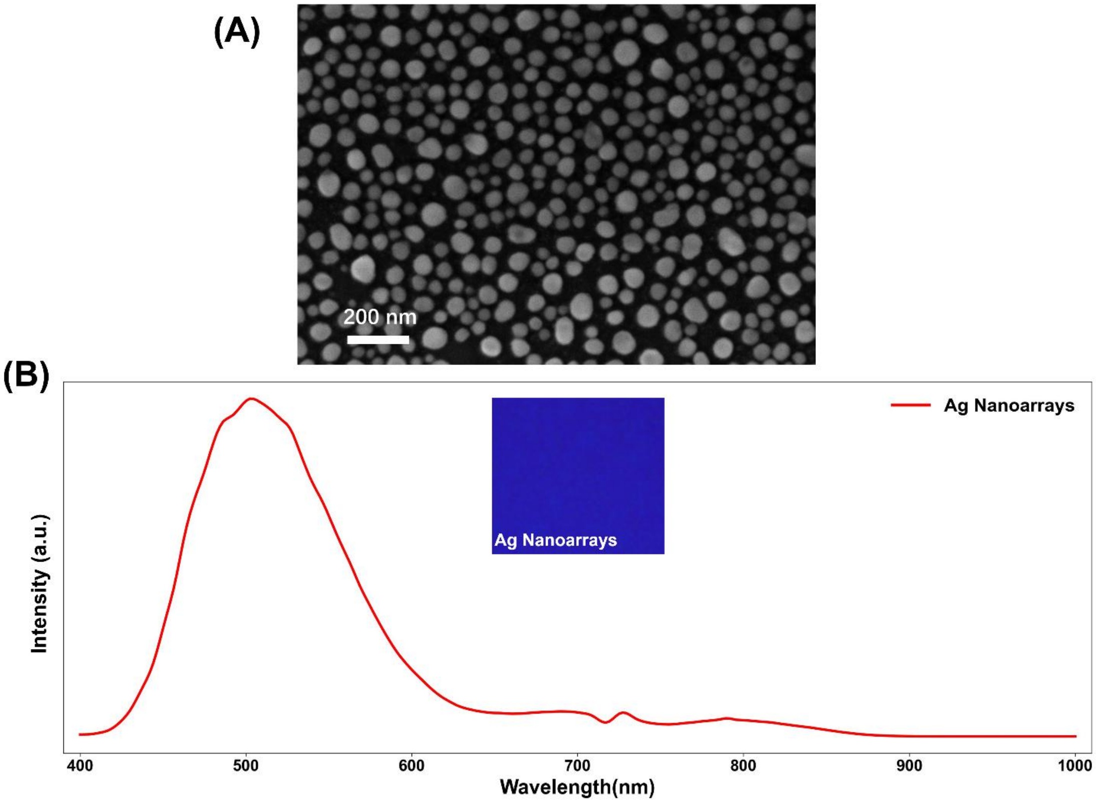

Figure S3. (A) The high-magnification SEM image and (B) micro-HSI reflection spectrum of the Ag nanoarrays after a 4-month interval.

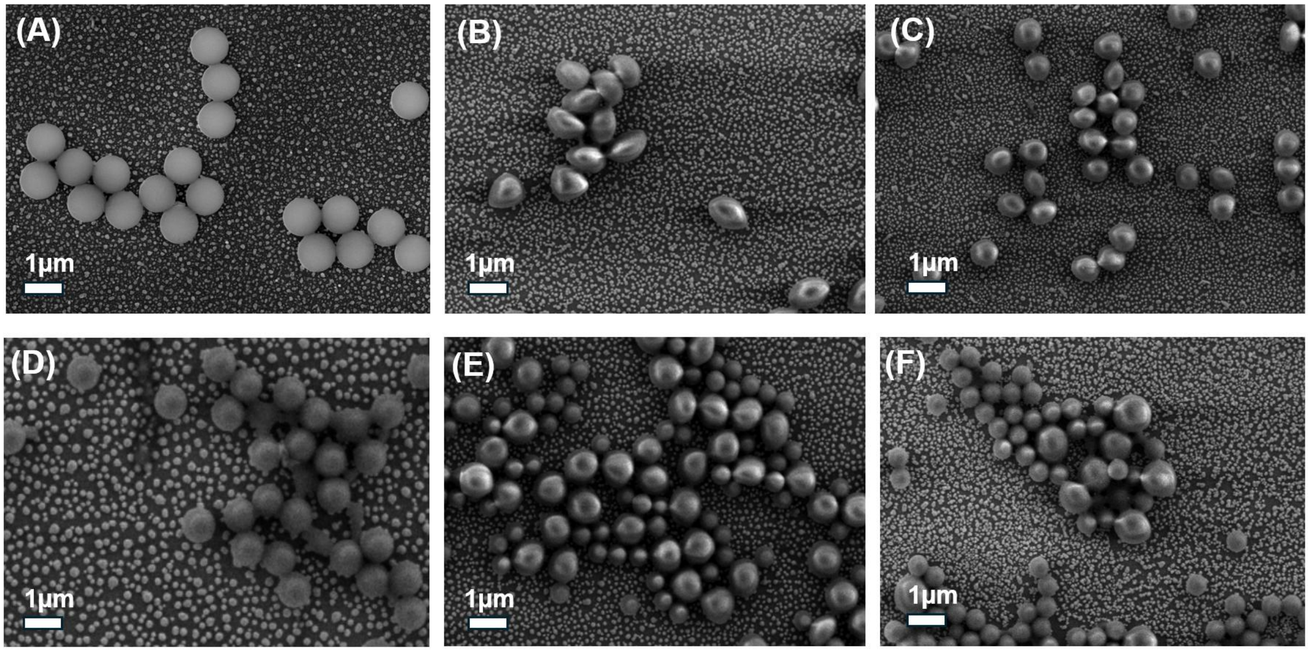

Figure S4. High-magnification SEM images of microplastic particles: (A) pure PS, (B) pure PET, (C) pure PMMA, and three mixed groups: (D) PS+PET, (E) PS+PMMA, and (F) PS+PET+PMMA.

As shown in the Figure S4, PS particles exhibit a uniform spherical morphology with an average size of approximately 1 μm. PET particles display a relatively irregular ellipsoidal shape, also with an average size around 1 μm. The PMMA samples have either spherical or ellipsoidal shapes, with sizes ranging from 0.5 to 1 μm. The three mixed samples show a more varied size distribution.

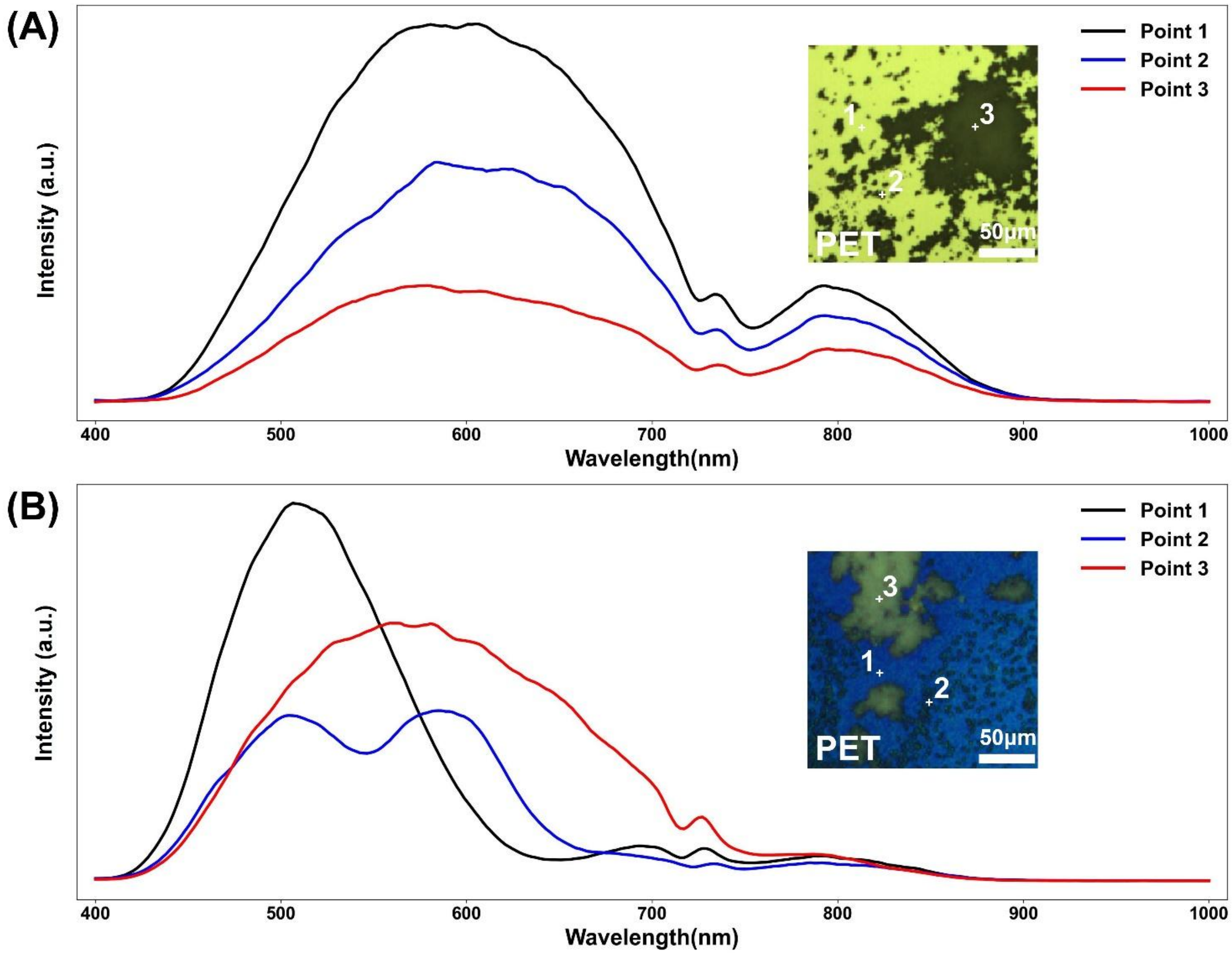

Figure S5. The micro-HSI reflection spectra of three representative points from dried PET microplastic samples on Si substrates (A) and Ag nanoarrays substrates (B). The insets in (A) and (B) provide 500× morphological images of the dried PET particles on Si and Ag nanoarray substrates. Points 1, 2, and 3 correspond to regions with low, moderate, and high particle densities, respectively.

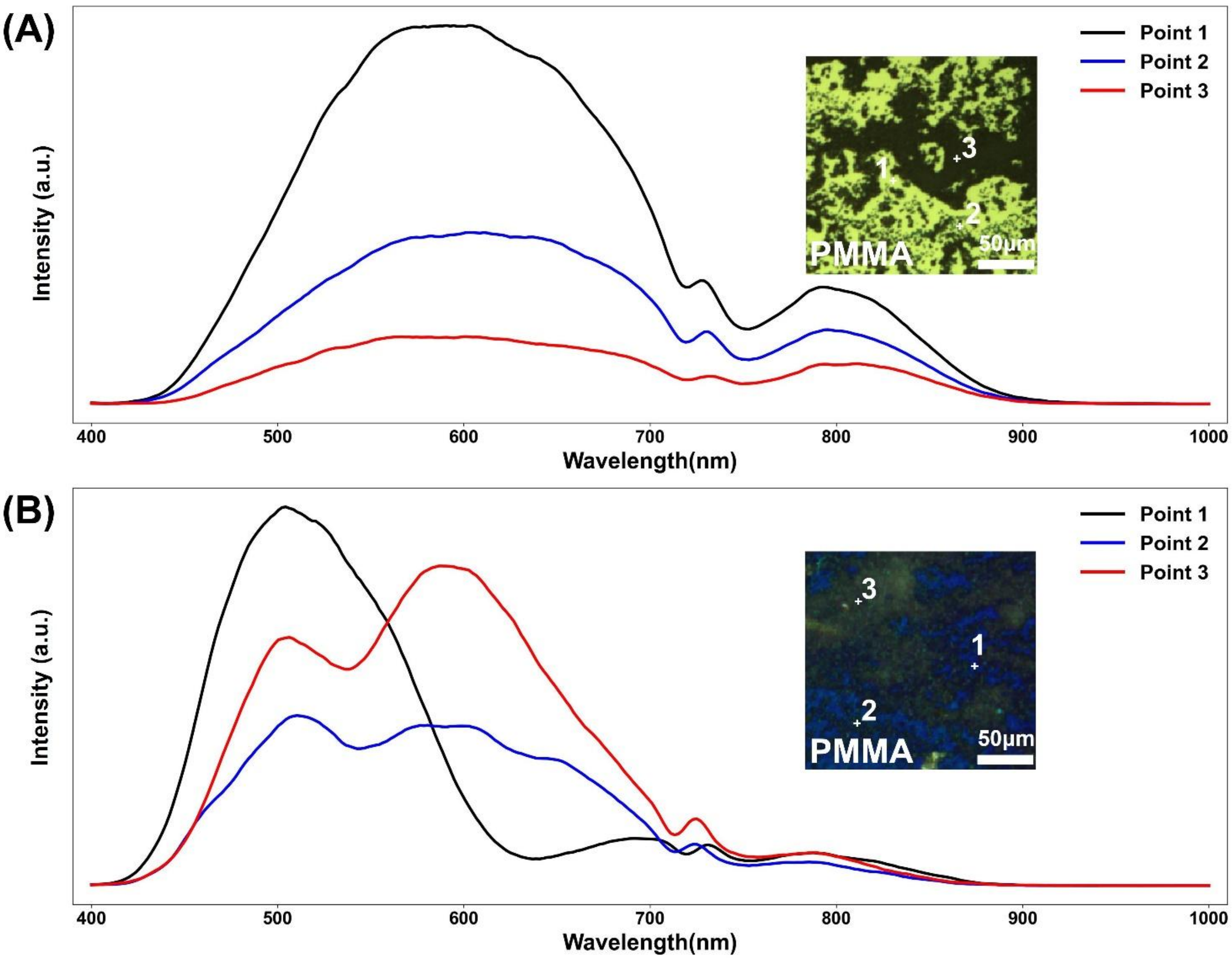

Figure S6. The micro-HSI reflection spectra of three representative points from dried PMMA microplastic samples on Si substrates (A) and Ag nanoarrays substrates (B). The insets in (A) and (B) provide 500× morphological images of the dried PMMA particles on Si and Ag nanoarray substrates. Points 1, 2, and 3 correspond to regions with low, moderate, and high particle densities, respectively.

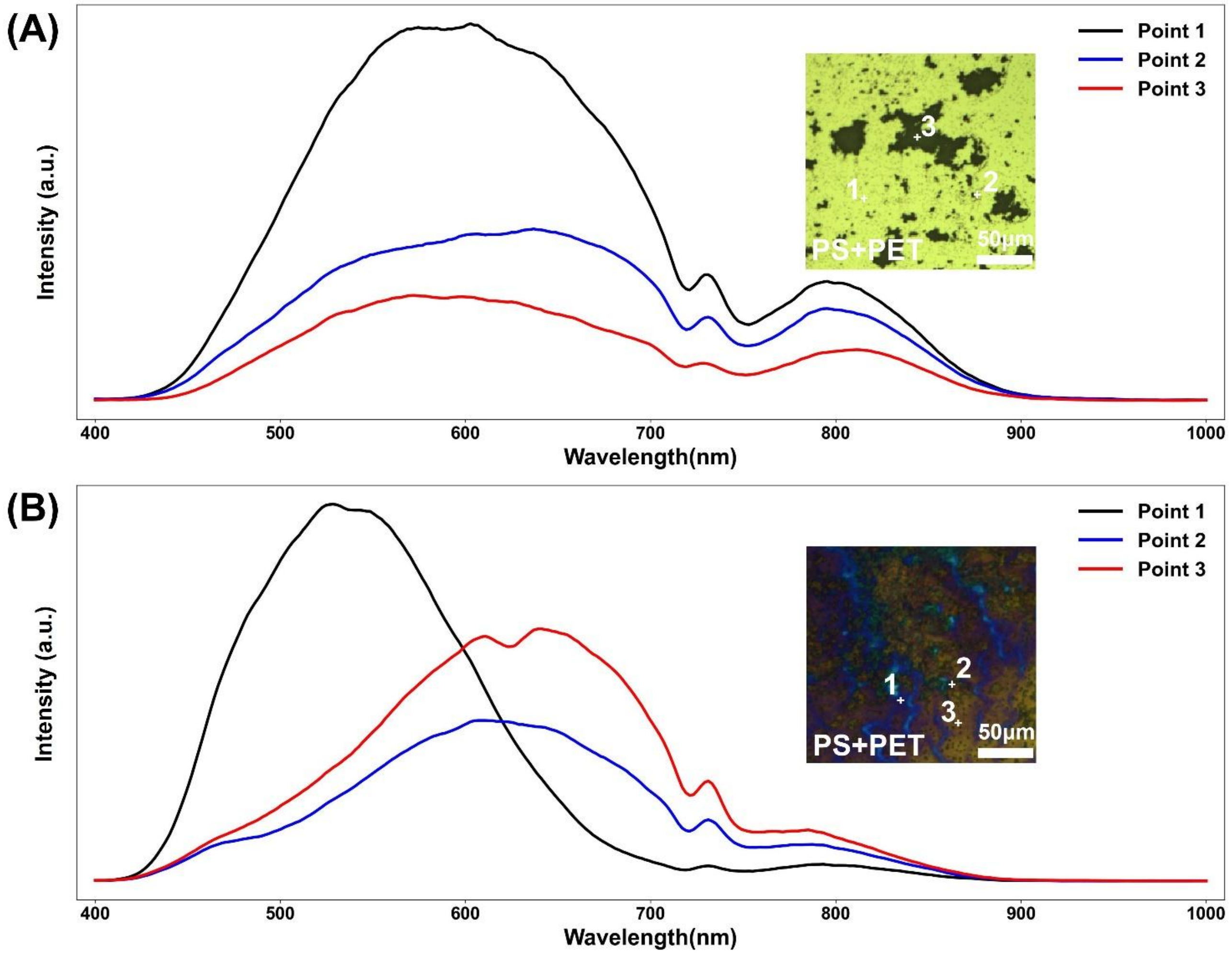

Figure S7. The micro-HSI reflection spectra of three representative points from dried PS+PET microplastic samples on Si substrates (A) and Ag nanoarrays substrates (B). The insets in (A) and (B) provide 500× morphological images of the dried PS+PET particles on Si and Ag nanoarray substrates. Points 1, 2, and 3 correspond to regions with low, moderate, and high particle densities, respectively.

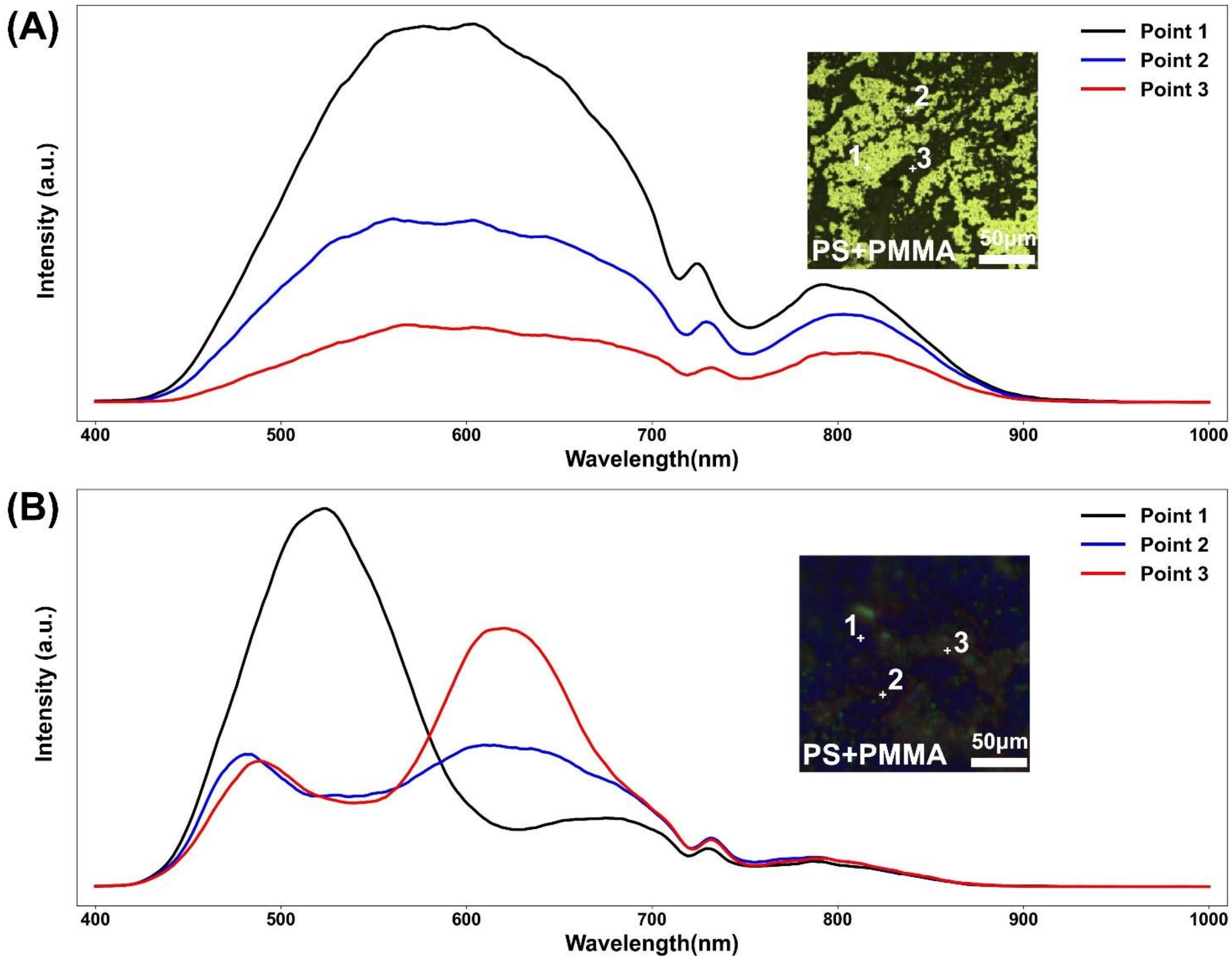

Figure S8. The micro-HSI reflection spectra of three representative points from dried PS+PMMA microplastic samples on Si substrates (A) and Ag nanoarrays substrates (B). The insets in (A) and (B) provide 500× morphological images of the dried PS+PMMA particles on Si and Ag nanoarray substrates. Points 1, 2, and 3 correspond to regions with low, moderate, and high particle densities, respectively.

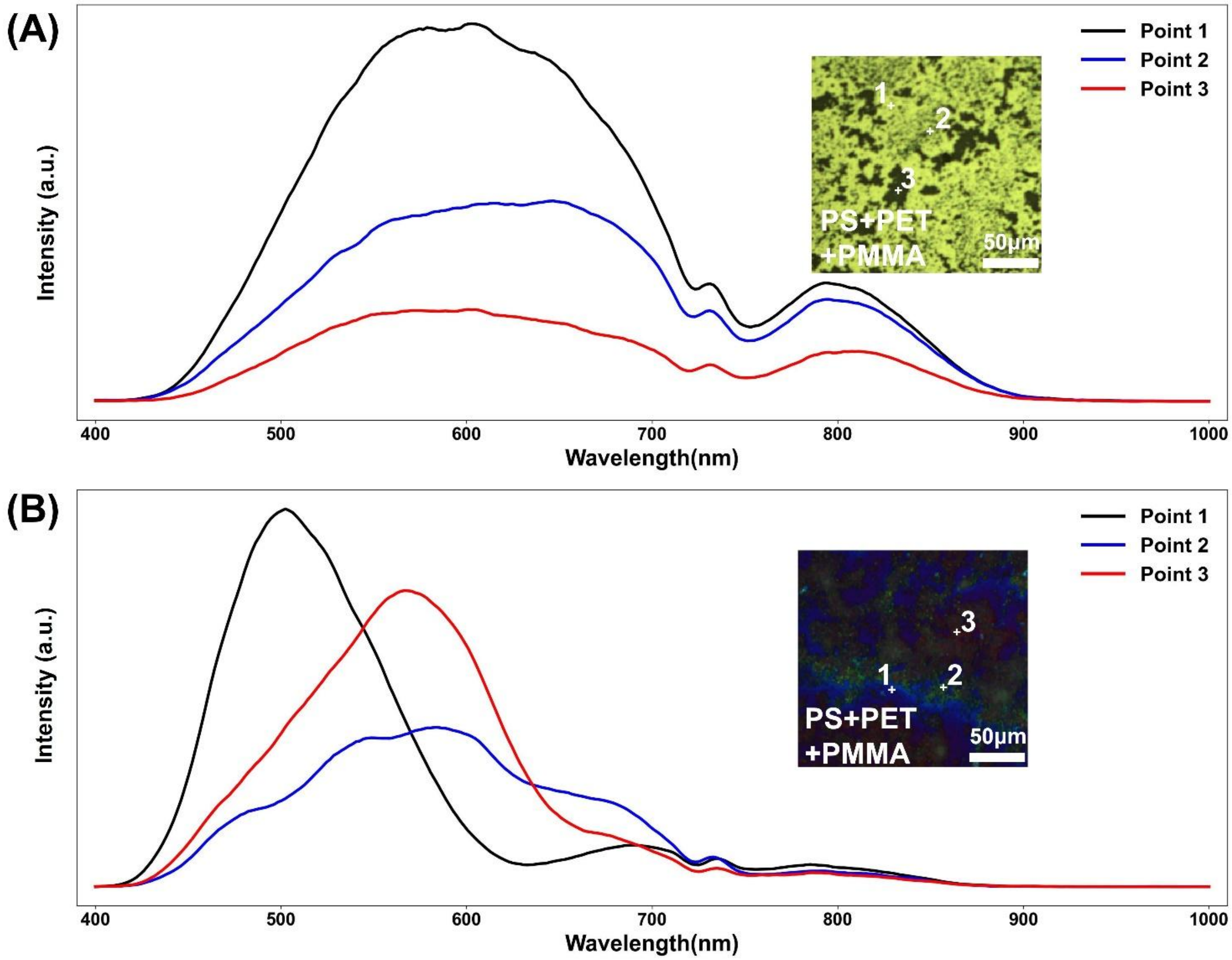

Figure S9. The micro-HSI reflection spectra of three representative points from dried PS+PET+PMMA microplastic samples on Si substrates (A) and Ag nanoarrays substrates (B). The insets in (A) and (B) provide 500× morphological images of the dried PS+PET+PMMA particles on Si and Ag nanoarray substrates. Points 1, 2, and 3 correspond to regions with low, moderate, and high particle densities, respectively.

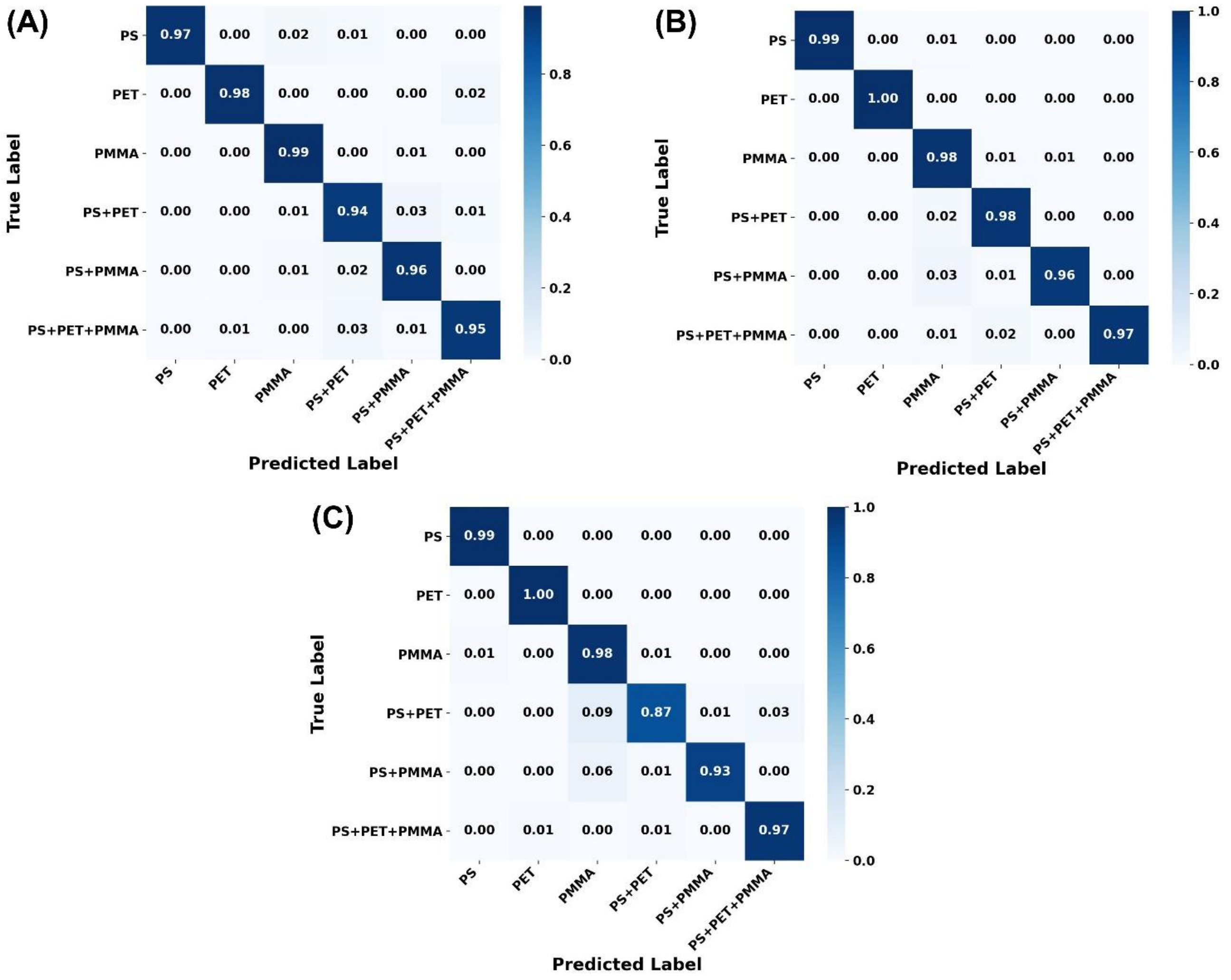

Figure S10. Confusion matrices for different models applied on Ag nanoarray substrates. (A) Confusion matrix of the 3D convolution-fully connected layer model. (B) Confusion matrix of the 2D convolution-fully connected layer model. (C) Confusion matrix of the fully connected layer model; Due to the enhanced effect of Ag nanoarray substrates, these models demonstrate solid performance; however, their accuracy consistently falls short of our proposed hybrid 3D-2D CNN model.

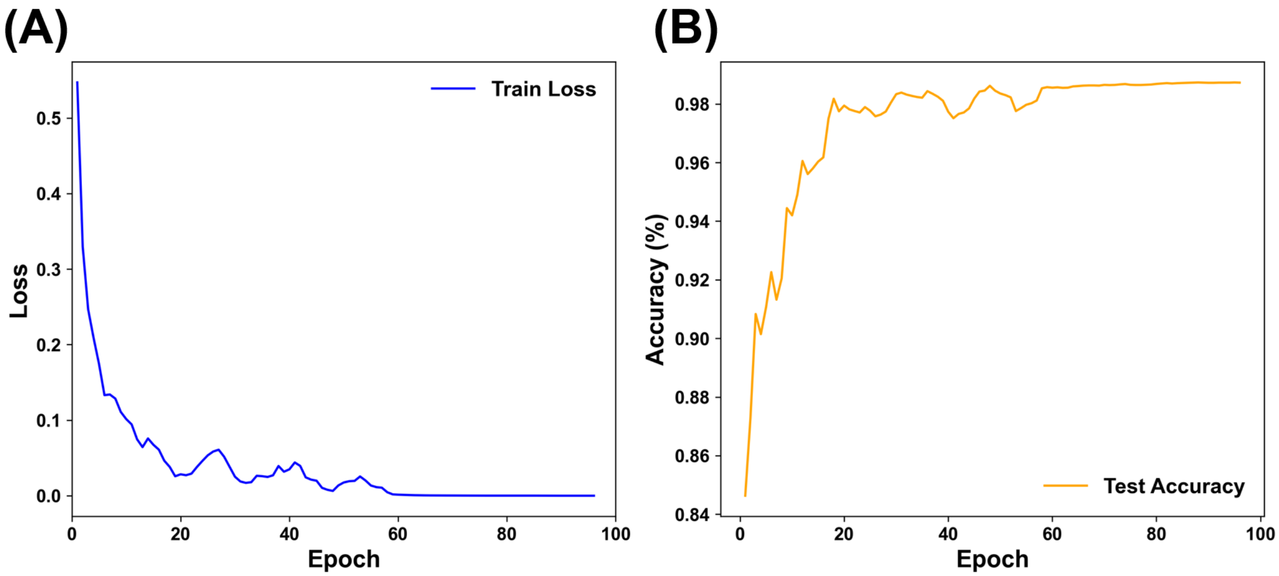

Figure S11. (A) Training loss curve and (B) Test accuracy curve of the 3D-2D CNN model applied to Micro-HSI datasets obtained from Ag nanoarray substrate.

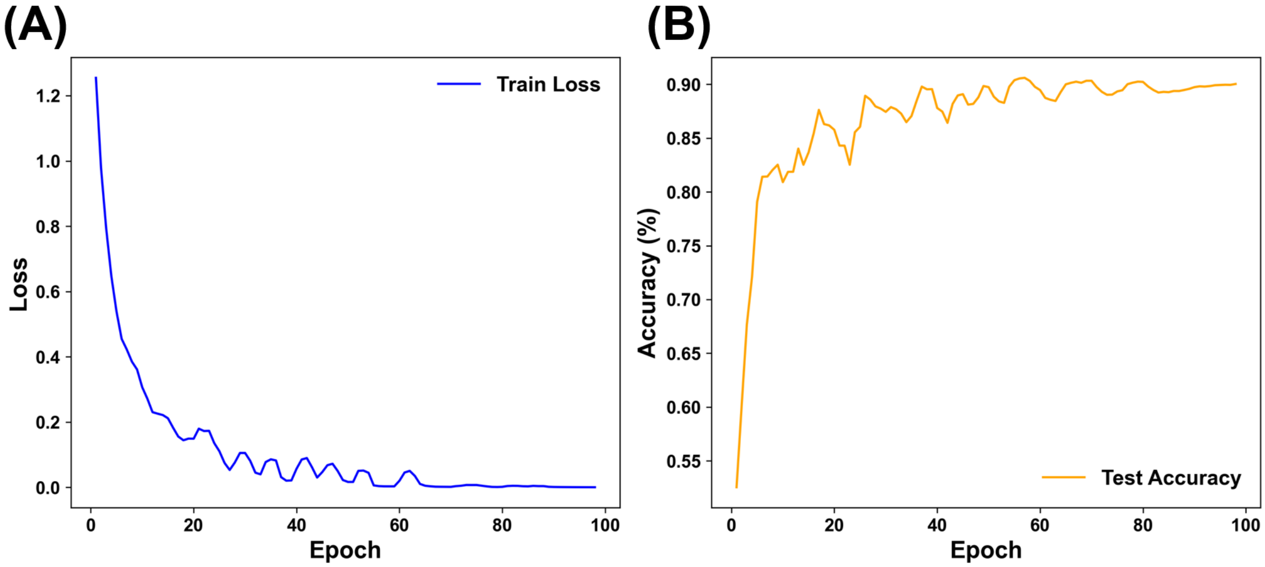

Figure S12. (A) Training loss curve and (B) Test accuracy curve of the 3D-2D CNN model applied to Micro-HSI datasets obtained from Si substrates.

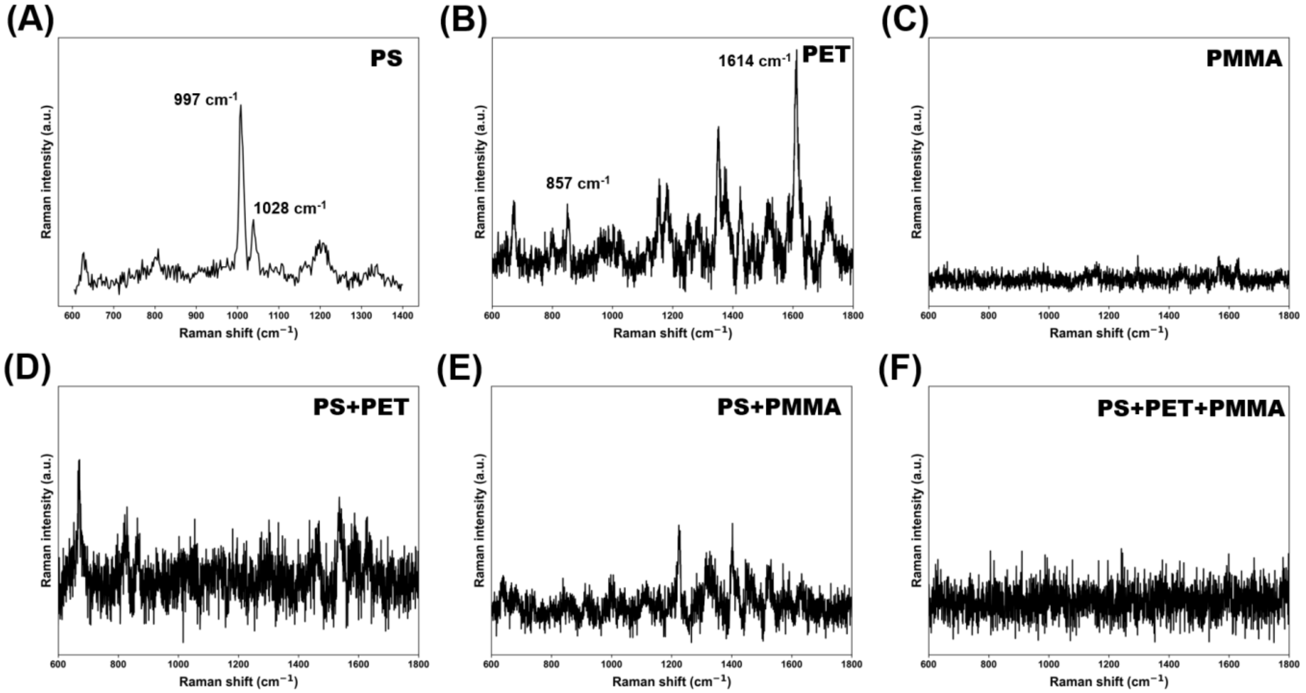

Figure S13. Raman spectra of microplastic particles: (A) pure PS, (B) pure PET, (C) pure PMMA, and three mixed groups: (D) PS+PET, (E) PS+PMMA, and (F) PS+PET+PMMA.

Our results indicate that, while Raman spectroscopy detected relatively weak signals for PS and PET samples, it only identified characteristic peaks at 997 and 1028 cm^{-1} for PS, and at 857 and 1614 cm^{-1} for PET.(Scientific Reports, 2022, 12: 18785) It struggled to reveal distinct signal peaks for other microplastic types. Based on the SEM images of the microplastics, the PMMA samples appeared to have slightly smaller particles, which may explain why they were more difficult to detect. Additionally, no effective signals were detected for the mixed sample, further demonstrating the limitations of Raman spectroscopy in detecting microplastics across the six samples of this study.

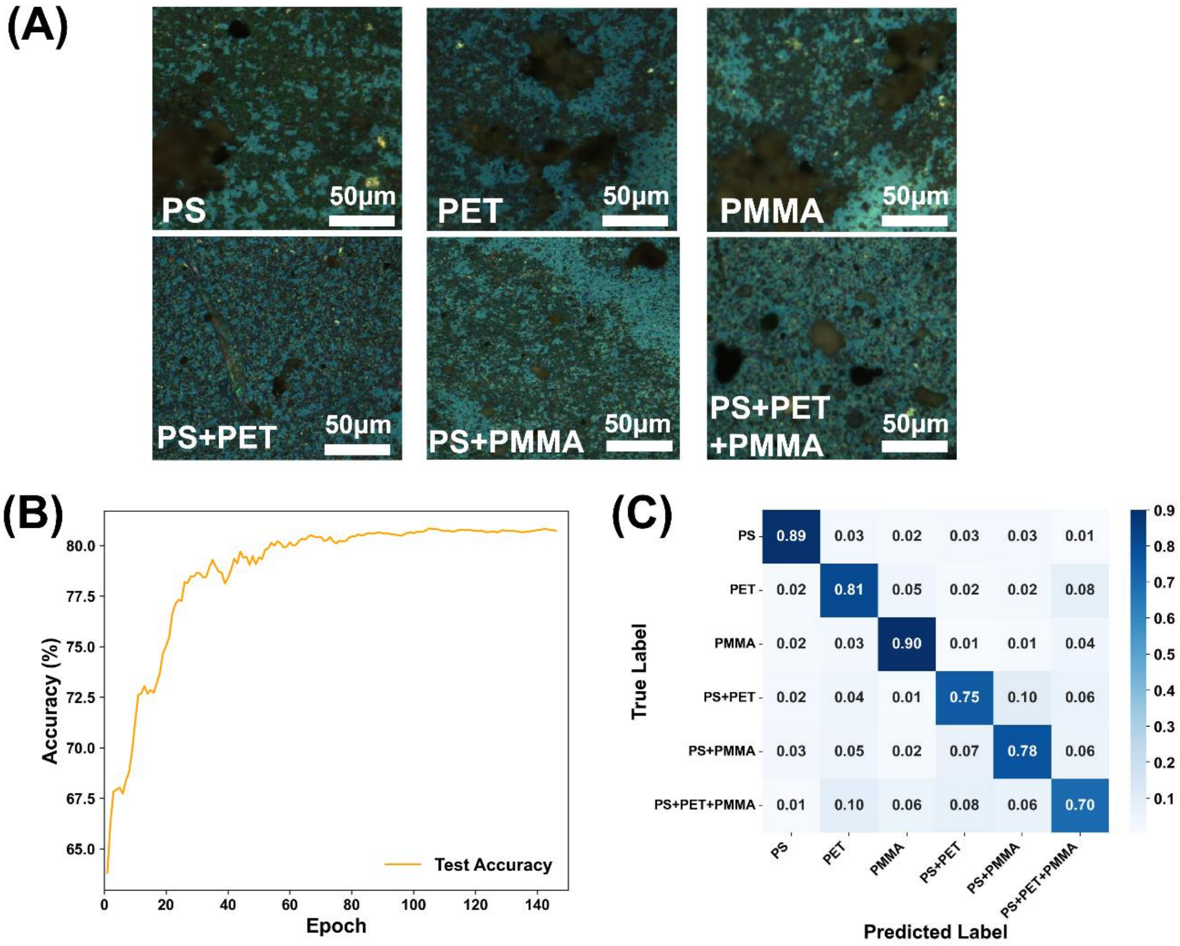

Figure S14. (A) 500× magnification morphological images of dried microplastic particles derived from six river water samples, including pure PS, PET, PMMA, and three mixed groups (PS+PET, PS+PMMA, and PS+PET+PMMA), deposited on Ag nanoarray substrates. (B) Test accuracy curve and (C) confusion matrix of the 3D-2D CNN model applied to Micro-HSI datasets of river water microplastics deposited on Ag nanoarray substrates.

As illustrated in Figure S14, Figure S14A presents the pseudo-three-channel color images of six microplastic samples dried after being exposed to river water. It is evident from these images that various impurities of different sizes, introduced by the river water, are present. Figure S14C shows the confusion matrix results, which indicate that the classification performance significantly declined due to the interference from environmental impurities of varying sizes. The overall accuracy of the 3D-2D CNN model decreased to 80.64%, a substantial drop from 98.98% observed in purified environments. Additionally, the recall, F1 score, and precision were found to be 80.64%, 80.58%, and 80.57%, respectively.
